# Modifiers of the Dipole Potential of Lipid Bilayers

**Published:** 2015

**Authors:** S. S. Efimova, O. S. Ostroumova

**Affiliations:** Institute of Cytology of the Russian Academy of Sciences, Tikhoretsky av. 4, St. Petersburg 194064 , Russia

**Keywords:** Dipole modifiers, membrane dipole potential, xanthene and styrylpyridinium dyes, muscle relaxants, planar lipid bilayers, spontaneous curvature, thyroid hormones, flavonoids

## Abstract

This paper assesses the magnitude of change in the dipole potential (φd) of membranes caused by the adsorption of modifiers on lipid bilayers of various compositions. We tested flavonoids, muscle relaxants, thyroid hormones, and xanthene and styrylpyridinium dyes in order to assess their dipole-modifying properties. A quantitative description of the modifying action of flavonoids, muscle relaxants, thyroid hormones, and xanthene dyes is shown as the ratio of the maximum change in the bilayer dipole potential upon saturation and the absolute φ_d_ value of the unmodified membrane. The slopes of the linear relationship between the increase in the dipole potential of phospholipid bilayers and the concentration of styrylpyridinium dyes in membrane-bathing solutions were found. We described the relationships between the change in φd and the chemical structure of modifiers, as well as the charge and spontaneous curvature of lipid monolayers.

## INTRODUCTION

The finding of agents that are able to affect the value of the membrane dipole potential, φd, and hence to regulate the processes of transport through the plasma membrane both in health and disease is one of the most relevant problems of modern molecular pharmacology. This potential jump at the bilayer–solution interface occurs as a result of a certain relative orientation of the dipoles of membrane lipids and water molecules adsorbed on the bilayer surface [1-4]. The dipole potential of a membrane depends on its lipid composition. The essential role is played by the unsaturation, length, and number of hydrocarbon chains in phospholipid molecules [5-7]. The most common dipole modifiers are amphiphilic substances, whose molecules have a significant dipole moment and are characterized by a specific orientation on the phase interface. There exist published data of the successful use of dipole modifiers to study the molecular mechanisms of formation and functioning of ion channels formed by various toxins and antimicrobial agents [8-25]. It was found that dipole- modifying properties are characteristic of some flavonoids, steroids, thyroid hormones, and xanthene and styrylpyridinium dyes [1-3, 26-30].

Flavonoids are the most common phytogenic phenolic compounds. They are derivatives of benzo-gamma- pyrone, whose structure is based on the scaffold consisting of two benzene rings (A and B) interconnected by a three-carbon fragment (C_2_-C_3_-C_4_). Classification of flavonoids is based on the degree of oxidation of pyran (2-phenylchromane or C-ring). The following groups are recognized: chalcones, flavanones, flavonols, flavanones, flavanonoles, iso-flavonoids, and others. Until recently, it was believed that the magnitude of membrane dipole potential can be affected only by chalcones, phloretin, and its glycoside, phlorizin [1, 3, 31].

Muscle relaxants are used to reduce the tone of skeletal muscles, including complete immobilization. Ammonium steroids (vecuronium, pancuronium, and rocuronium) are non-depolarizing relaxants. The structure of muscle relaxants is based on the steroid nucleus. There exist published data on the impact of some steroids on φ_d_. Thus, it has been shown [32] that the introduction of cholesterol, 6-ketocholestanol, or a coprostanol in the membrane-forming solution of dimyristoylphosphocholine results in increased membrane dipole potential. Extraction of 5α-androstan-3β-ol from the lipid bilayer leads to an increase in membrane conductance induced by K^+^-nonactin [20]. This result indicates that 5α-androstan-3β-ol enhances the dipole potential of the bilayer. It was found [33] that the steroid hormone pregnenolone reduces the dipole potential of liposome membranes formed of a mixture of dipalmitoylphosphatidylcholine and cholesterol.

Thyroid hormones thyroxine and triiodothyronine play a key role in metabolism regulation. They are iodinated tyrosine derivatives and differ from each other in the number and location of iodine atoms. It was also shown that iodine-containing thyroid hormones, as well as flavonoid phloretin, reduce the dipole potential of cholesterol-containing lipid bilayers [27].

Xanthene dyes are represented by two groups: fluoresceins and rhodamines. The first group includes fluorescein and its halogen derivatives (erythrosin, eosin, Rose Bengal, and phloxine B). The second group includes xanthene dyes that belong to the rhodamine family. They are fluorescein derivatives with both hydroxyl groups replaced by alkylated amino groups. It was shown that adsorption of Rose Bengal on the surface of the diphytanoylphosphatidylcholine membrane leads to a decrease in the membrane dipole potential [29], similarly to the effect of phloretin.

Styrylpyridinium dyes that belong to the ANEPPS and RH series are potential-sensitive fluorochromes based on styrylhemicyanines. They differ in the length of their hydrocarbon tails and (or) polyene fragment. These fluorescent dyes have a high dipole moment due to a delocalized positive charge in the pyridine complex and negative charge of the sulfogroup at the other end of the molecule. RH dyes enhance the φ_d_ of phosphocholine membranes, and this ability decreases in the order RH 421, RH 237, and RH 160 [28].

The aim of our study was assessing and quantifying the effect of certain flavonoids, muscle relaxants, thyroid hormones, and fluorescent dyes on the dipole potential of lipid bilayers of various compositions. Particular attention was paid to the relationship between the chemical structure of modifiers and the dipolemodifying effectiveness of these compounds.

## EXPERIMENTAL

**Materials**

The following reagents were used: KCl, HEPES, pentane, ethanol, chloroform, dimethylsulfoxide (DMSO), hexadecane, and squalene (Sigma, USA); phloretin, phlorizin, rutin, genistin, genistein, quercetin, myricetin, biochanin A, (±) catechin hydrate, (±) taxifolin hydrate, daidzein, 2’,4’,6’-trihydroxyacetophenone monohydrate (THAP), 2’-hydroxy-4’,6’-dimethoxyacetophenone (DHAP), RH 421, di-8-ANEPPS, L-thyroxine, 3,3’,5’-triiodo-L-thyronine, Rose Bengal, phloxineB, erythrosin, eosin Y, fluorescein, rhodamine 6G, rhodamine 101, pancuronium bromide, vecuronium bromide, and rocuronium bromide (Sigma, USA); RH 160 and RH 237 (Molecular Probes, USA);1,2-diphytanoyl- sn-glycero-3-phosphocholine (DPhPC), 1,2-dioleoyl-sn-glycero-3-phosphocholine (DOPC), 1,2-dioleoyl-sn-glycero-3-phosphoserine (DOPS), 1,2-dioleoyl-sn-glycero-3-phosphoethanolamine (DOPE) (Avanti Polar Lipids, USA). The chemical structures of the used modifiers are shown in *Table 1*.

**Table 1:** The relative changes in the dipole potential of DPhPC membranes in the presence of various modifiers (γ) and their dissociation constants (K)

Class	Dipole modifier	Chemical structure	γ, %	K, μM
Flavonoids	Phloretin	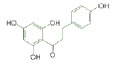	-59 ± 12	2.0 ± 0.5*
	Phlorizin	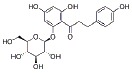	-37 ± 7	5.1 ± 0.2*
	Quercetin	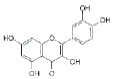	-42 ± 9	3.3 ± 0.5*
	Myricetin	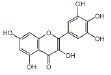	-44 ± 12	3.3 ± 0.2*
	Ruthin	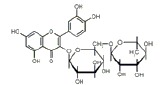	-17 ± 5	10.8 ± 0.5
	Biochanin A	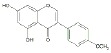	-37 ± 10	2.1 ± 0.3*
	Daidzein	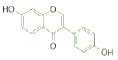	-8 ± 4	8.8 ± 0.2
	Genistein	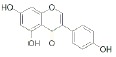	-28 ± 8	1.3 ± 0.2*
	Genistin	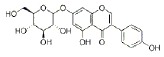	-2 ± 2	9.6 ± 0.5
	Catechin	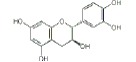	-1 ± 1	0.7 ± 0.2
	Taxifolin	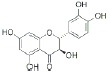	-3 ± 1	5.8 ± 0.6
	THAP	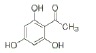	-6 ± 3	26.4 ± 5.6*
	DHAP	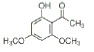	-20 ± 5	10.2 ± 0.4
Muscle relaxants	Pancuronium	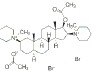	2 ± 2	0.1 ± 0.1
	Vecuronium	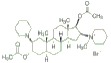	1 ± 1	0.1 ± 0.1
	Rocuronium	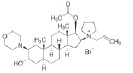	2 ± 2	0.1 ± 0.1
Thyroid hormones	Thyroxine	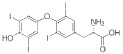	-24 ± 7	3.5 ± 0.1@
	Triiodothyronine	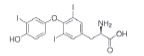	-23 ± 7	5.3 ± 0.2@
Xanthene dyes	Fluorescein	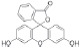	-2 ± 1	0.4 ± 0.1@
	Eosin Y	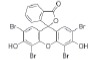	-2 ± 1	0.4 ± 0.1@
	Erythrosin	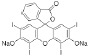	-26 ± 7	0.8 ± 0.1@
	Rose Bengal	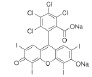	-48 ± 11	0.2 ± 0.1@
	Phloxine B	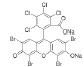	-33 ± 7	0.2 ± 0.1@
	Rhodamine 101	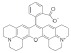	-9 ± 4	0.3 ± 0.1
	Rhodamine 6G	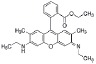	-4 ± 1	0.4 ± 0.1
Styrylpiridinium dyes	RH 160	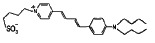	15 ± 6	-
	RH 237	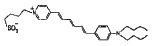	19 ± 4	-
	RH 421	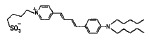	47 ± 9	-
	di-8-ANEPPS	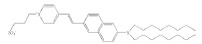	1 ± 1	-

@ Results are taken from [44].

* Results are taken from [46].

**Measurements of the change in the dipole potential of lipid bilayers**

The bilayer lipid membranes were formed using the Montal and Mueller method [34] by combining condensed lipid monolayers in a hole in the Teflon film that separated the experimental chamber into two*(cis- *and *trans-) *compartments. The volume of each compartment was 1.5 ml, Teflon film thickness was 10 μm, and hole diameter was about 50 μm. Before the beginning of the process, the hole in the Teflon membrane was pretreated with hexadecane. Monolayers were formed on the water–air interface using a 1 or 2 mg/ml lipid solution in pentane. DPhPC, DOPC, DOPE, DOPS, and an equimolar mixture of DOPE and DOPS (DOPS/DOPE) were used to obtain the monolayers. The experiments were performed using the same ionic composition of aqueous solutions of electrolyte in both compartments of the chamber (0.1 M KCl). Solution acidity (pH 7.4) was maintained with a 5 mM HEPES–KOH buffer.

Ionophores nonactin or valinomycin in the form of an ethanol (7 mg/ml) or methanol (0.8 mg/ml) solution, respectively, was added to the aqueous phase of the two compartments of the chamber to a final concentration of 10^-7^-10^-5^ M. It is known that phloretin in phospholipid membranes is less effective with respect to the transmembrane current induced by K^+^-valinomycin compared to that induced by K^+^-nonactin [1]. Similar results were obtained in preliminary experiments with other flavonoids, as well as muscle relaxants, xanthene dyes, and thyroid hormones. For this reason, changes in the membrane dipole potential caused by the introduction of these modifiers were measured using nonactin. It was found that styrylpyridinium dyes in the DPhPC bilayer are less effective with respect to the transmembrane current induced by K^+^-nonactin compared to that induced by K^+^-valinomycin. For this reason, valinomycin was used in the experiments measuring the increase in the membrane dipole potential caused by adsorption of these dyes.

Modifiers were added to both compartments of the chamber from millimolar solutions in ethanol, DMSO, or water to final concentrations in the membrane bathing solutions of 2.5 to 150 μM for flavonoids, 1 μM to 1 mM for muscle relaxants, 0.25 to 10 μM for xanthene dyes, 1 to 50 μM for thyroid hormones, and 1 to 10 μM for styrylpyridinium dyes.

The final concentration of the solvent (ethanol, methanol, or DMSO) in the chamber did not exceed 0.1%. This concentration of the above-mentioned solvents did not cause the integrity and stability of the lipid bilayers. In the absence of ionophores, dipole modifiers at maximum concentrations likewise did not affect the conductance of the model membranes.

Transmembrane currents were measured and digitized in the voltage clamp mode, using Axopatch 200B and Digidata 1440A (Axon Instruments, USA). Silver– silver chloride electrodes (Ag/AgCl) connected to the solutions in the chamber through bridges with 1.5% agarose in the 2 M solution of KCl were used to apply the transmembrane potential (V) and record signals from the membrane. Measurements were performed at room temperature.

The data were processed using an 8-pole Bessel filter (Model 9002, Frequency Devices) and a filtering frequency of 1 kHz. Transmembrane current recordings were processed using the Clampfit 9.0 software package (Axon Instruments, USA). Statistical analysis of data was performed using the Origin 8.0 program (OriginLab, USA).

Membrane conductance (*G*) was determined as the ratio of the steady-state transmembrane current flowing through the lipid bilayer membrane (*I*) to the transmembrane potential (*V*), which was 50 mV. Change in the membrane dipole potential (Δφ_d_) caused by the introduction of the modifiers was assessed using Boltzmann statistics:

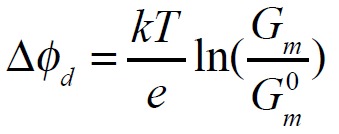



where *G^0^_m_*and *G_m_*are the values of the steady-state K^+^-conductance of the bilayer induced by ionophore before and after the introduction of the modifier , *e *– electron charge, *k *– Boltzmann constant (1,38 x 10^-23^ J/K), and *T *– thermodynamic temperature (*T *= 294 K) [1].

The average values of the change in the membrane dipole potential were calculated as the arithmetic mean values of Δφ_d_ in each experimental system, measuring three to five bilayers (mean ± SD).

Adsorption of flavonoids, muscle relaxants, thyroid hormones, and xanthene dyes on the surface of the lipid bilayers was described using the Langmuir isotherm:

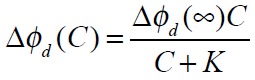



where Δφ_d_ (*C*) – change in the membrane dipole potential at the concentration (*C*) of the modifier in the membrane bathing solution; Δφ_d_ (∞) – maximum change in the membrane dipole potential at *C→∞; *and *K *– dissociation constant of the modifier that characterizes its affinity to the lipid phase [3, 26]. The Δφ_d_ (∞) value was determined using the plot of Δφ_d_ (*C*) function as the mean value corresponding to saturation, i.e. constant of the membrane dipole potential upon further increase in the concentration of the modifier. The *K *value was found as the slope of the linear approximation of the relationship

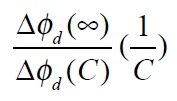



The error of Δφ_d_ (∞) was found as the maximum experimental error of the measurement of Δφ_d_ (*C*). The error of *K *was calculated as the error of the ratio:

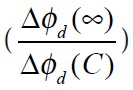



No saturation effect was observed within the measured concentrations of styrylpyridinium dyes (10 μM). Further increase of the concentration of the dye results in the destruction of the lipid bilayer. For these reasons, we used the expression resulting from the linearization of the equation (*2*) at low concentrations of the dipole modifier (*C* < < *K*) to describe the adsorption of styrylpyridinium dyes on the bilayer:

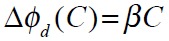



where

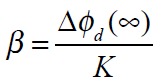



is the slope of the linear relationship between the increase in the bilayer dipole potential and dye concentration in the bathing solution [28].

The relative value of the change in the dipole potential (γ) was used to compare the effectiveness of the dipole-modifying action of various modifiers. It was calculated as follows:

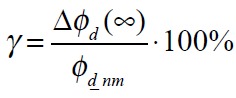



where φ_d_nm_ is the value of the dipole potential of the unmodified membrane, which can be found in the literature. In the absence of dipole modifiers, the dipole potential of DPhPC, DOPC, DOPS, and DOPE membranes was equal to 250 ± 40 [5, 35, 36], 225 ± 20 [5], 240 ± 20 mV [37, 38], and 220 ± 5 mV [5, 35, 36], respectively. φ_d_nm_ of DOPS/DOPE-bilayers was calculated as the average of φ_d_nm_ values for DOPS and DOPE membranes. The error of γ was calculated as the error of the ratio:

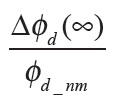



In the case of styrylpyridium dyes, the γ value was calculated as the ratio of the change in the bilayer dipole potential at 5 μM concentration of the modifier to φ_d_nm_. It was assumed that the agents having “weak” dipole-modifying properties are characterized by a γ value ranging from 0 to 10 %, “average”– 10 to 30 %, and “strong”– 30 to 60 %.

## RESULTS AND DISCUSSION

**Flavonoids**

It is known that adsorption of phloretin on the membrane in a first approximation can be described by the Langmuir adsorption isotherm (1), which is characterized by the following: maximum change in the dipole potential upon saturation (Δφ_d_ (∞)) and dissociation constant of the flavonoid (*K*) [3, 39]. *Table 1* shows γ values (4) that characterize the relative change in the membrane dipole potential at the introduction of various modifiers. As shown from *Table 1*, all flavonoids reduce the membrane dipole potential. All the agents under study can be conveniently divided into three groups according to the intensity of the dipole-modifying properties. The first group includes modifiers with “low” effectiveness, which have a γ value ranging from 0 to 10 %. These are isoflavonoids daidzein and genistin, flavanol catechin, flavanonol taxifolin, and synthetic phloroglucinol THAP. The second group includes compounds having more pronounced dipole-modifying properties, so-called agents with “average”-effectiveness (γ ranges from 10 to 30 %). They are flavonol rutin, isoflavone genistein, and phloroglucinol DHAP. The third group includes the “strongest” flavonoid dipole modifiers characterized by a γ value of 30 to 60 %. They are chalcones phloretin and phlorizin, flavonols quercetin and myricetin, and isoflavone biochanin A.

When comparing the chemical structures of the flavonoids shown in *Table 1*, one can conclude that the dipole-modifying properties of modifiers are associated with the presence of a double bond in the C-ring of flavonoid molecules. There is no double bond in the C-rings of taxifolin or catechin as opposed to that of quercetin. As a result, bent-shaped flavanol and flavanonol have practically no influence on the φ_d_ value, while adsorption of planar flavonol on the membrane leads to a significant decrease in the dipole potential of the latter. When analyzing *Table 1*, it can also be seen that a higher γ value correlates with a lower number of hydroxyl groups in the flavonoid molecule. Thus, the decrease in φ_d_ caused by the adsorption of phlorizin (phloretin glycoside) is less pronounced compared to that in the case of the more hydrophobic aglycone phloretin. A similar situation is observed for flavonols (quercetin/myricetin and rutin), isoflavones (biochanin A, genistein, and genistin), and phloroglucinols (DHAP and THAP). Unlike biochanin A, isoflavone daidzein has practically no effect on the value of the dipole potential of DPhPC membranes, despite its small number of hydroxyl groups. Since the dissociation constant of daidzein is higher compared to that of biochanin A, it can be assumed that the former has lower affinity to the lipid phase compared to the latter. The observed differences can also be caused by the different orientations of daidzein and biochanin A in the membrane due to the fact that the daidzein molecule has two hydroxyl groups located on opposite sides of the molecule, while in the biochanin A molecule they are located on the same side. It should also be noted that the dissociation constants of glycosides (phlorizin, rutin, and genistin) surpass this parameter in the corresponding aglycones (phloretin, quercetin, and genistein). It is likely that this fact, as well as the less pronounced variation of φ_d_ in the presence of glycosides compared to that in the presence of aglycones, is due to the greater hydrophilicity of glycosides, and hence their lower affinity to the lipid phase. Phloroglucinols THAP and DHAP have the highest desorption constants among the studied flavonoids. The latter observation is consistent with the results of [40], which showed that the distribution coefficient of THAP between lecithin and water is 8 times lower than that of phloretin.

**Table 2:** The relative changes in the dipole potential of phospholipid bilayers in the presence of various modifiers (γ) and their dissociation constants (K)

Dipole modifier	Parameter	DOPC	DOPE	DOPS	DOPS/DOPE (50/50 mol.%)
Phloretin	γ, %	-62 ± 9	-58 ± 5	-38 ± 6	-41 ± 8
	K, μM	0.7 ± 0.2*	2.2 ± 0.4*	2.7 ± 0.8*	2.8 ± 0.2
Thyroxine	γ, %	-18 ± 5	-25 ± 4	-16 ± 5	-22 ± 6
	K, μM	3.8 ± 0.3	5.9 ± 0.5	2.8 ± 0.3	3.4 ± 0.2
RH 421	γ, %	27 ± 5	25 ± 2	5 ± 2	25 ± 9

* Results are taken from [46].

*Table 2* shows the characteristic parameters of the adsorption isotherm of the “strongest” flavonoid modifier, phloretin, on lipid bilayers of different compositions. As *Table 2* shows, the ability of phloretin to reduce φd depends on the type of membrane-forming lipid. DOPE (because it is unsaturated) and DPhPC (because it has branched hydrocarbon chains) tend to form non-lamellar structures; so the bilayers formed of these lipids are characterized by elastic tension due to the deformation of monolayers having a negative spontaneous curvature. This tension can be detected when investigating the profile of the lateral pressure in the bilayer [41, 42]. DOPC forms a lamellar structure, and monolayers that contain DOPC have a very low spontaneous curvature. The maximum decrease in the dipole potential caused by the adsorption of phloretin is practically the same for DOPC, DOPE, and DPhPC membranes (*Table 1* and *Table 2*). These results indicate that the plane of adsorption of the modifier in the membrane does not match the plane of the lateral pressure jump in DOPE and DPhPC bilayers. Furthermore, phloretin is about 1.5 times less effective with respect to bilayers comprising negatively charged DOPS phospholipid (DOPS or DOPS/DOPE) compared to membranes formed of uncharged phospholipids (DPhPC, DOPC, or DOPE). A similar result was obtained [43] in the study of phloretin adsorption on neutral and negatively charged monolayers consisting of dimyristoylphosphatidylcholine and dimyristoylphosphatidylglycerol, respectively. Taking into account that the uncharged form of phloretin [1] is responsible for the decrease in the dipole potential, the observed differences cannot be determined by the decreased adsorption of the charged form of the modifier on DOPS-containing membranes. This is also evidenced by the close values of phloretin dissociation constants for uncharged DPhPC, DOPC, and DOPE and charged DOPS membranes (*Tables 1 *and *2*). This suggests that the distribution ratio of the modifier is practically independent of the phospholipid composition of the membrane. This effect may stem from the positive spontaneous curvature of DOPS monolayers, arising from the “repulsion” of negatively charged lipid heads. As a result, phloretin molecules adsorp tion in this region may acquire an orientation different from that in the uncharged membranes and/or have a greater number of possible conformations.

**Muscle relaxants**

As *Table1 *shows*, *addition of pancuronium bromide, vecuronium bromide, or rocuronium bromide to solutions bathing the DPhPC membrane has practically no effect on their dipole potential (γ value does not exceed 2 %). We can conclude that all the muscle relaxants under study have weak dipole-modifying properties. Given that a saturated steroid 5α-androstane-3β-ol that has only one hydroxyl group enhances the dipole potential of the lipid bilayers [20], it can be suggested that the lack of impact by steroid relaxants on φ_d_ is due to modifications that enhance the hydrophilicity of the steroid molecule (additional acetate groups and nitrogencontaining heterocycles). High hydrophilicity and the presence of functional groups at different ends of the pancuronium, vecuronium, and rocuronium molecules suggest that muscle relaxants adsorbed on the membrane surface are only slightly buried into the bilayer. A small depth of immersion is indirectly evidenced by the lack of effect by pancuronium bromide on the dipole potential of DOPC membranes (γ value is equal 3 ± 1), which, unlike DPhPC bilayers, do not have a lateral pressure jump on the hydrocarbon area. At the same time, small values of the dissociation constants of muscle relaxants (*Table 1*) are indicative of a high coefficient of distribution of these compounds between the bilayer and aqueous solution.

The surface charge of the membrane significantly affects the absorption of steroid muscle relaxants. Pancuronium bromide and vecuronium bromide enhance the dipole potential of negatively charged DOPS/DOPE membranes (γ value is equal 17 ± 3 %), while more hydrophobic rocuronium bromide has almost no effect on the φ_d_ value of DOPS/DOPE-bilayers (γ value is equal about 1 %). The dependence of the effects on the charge of membrane-forming lipids suggests that charged forms of modifiers are responsible for the change in the dipole potential caused by the introduction of pancuronium bromide and vecuronium bromide. However, the dissociation constant of these muscle relaxants is two orders of magnitude higher and, therefore, affinity is lower in DOPS/DOPE bilayers compared to those in DPhPC membranes, which is indicative of the opposite. It is likely that the observed differences are not due to electrostatic interaction between the modifiers and the DOPS-containing membrane, but rather the positive spontaneous monolayer curvature, as in the case of phloretin. This suggests that in the DOPS/DOPE bilayer pancuronium and vecuronium are located near the repulsing, negatively charged serine heads.

**Thyroid hormones**

Comparing the γ value of thyroid hormones showed that thyroxine and triiodothyronine are dipole modifiers with “average” effectiveness that enhance the dipole potential of DPhPC membranes in a similar manner. Similar results were obtained previously [27]. This finding suggests that the presence of an additional iodine atom in the thyroxine molecule (compared to the triiodothyronine molecule) has little effect on the dipole moment of the modifier and its orientation in the bilayer.

**Fig. 1:**
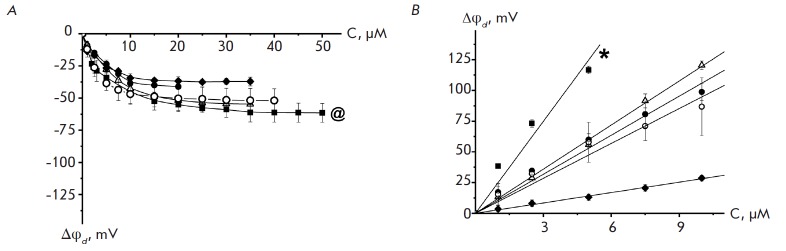
The dependence of change in the membrane dipole potential (Δφd) on concentration of thyroxine (A) and RH 421 (B) in the bathing solution. Membranes were made formed of DPhPC*,@ (■), DOPC (●), DOPS (♦), DOPE (Δ), and DOPS/ DOPE (50/50 mol. %) (o) and bathed in 0.1M solution of KCl at pH 7.4*.*V = 50 mV. @ Results are taken from [44]. * Results are taken from [46].

*Fig. 1A* shows the decrease in the dipole potential of DPhPC, DOPC, DOPS, DOPE, and DOPS/DOPE bilayers versus thyroxine concentration in the bathing solutions. *Table 2* shows γ values that characterize the relative changes in the membrane dipole potential caused by adsorption of thyroxine on lipid bilayers of different compositions. *Fig. 1A* and *Table 2* show that the effectiveness of thyroxine weakly depends on the charge of membrane-forming lipids. Similar results were obtained when comparing DPhPC and diphytanoylphosphoserine bilayers [44]. The lack of a relationship between the modifier effects (both the γ and *K *values) and membrane surface charge indicates that the decrease in the membrane dipole potential is caused by the adsorption of the uncharged form of iodine-containing thyroid hormones. This finding is also evidenced by data reported in [27]. The closeness of γ values for the DPhPC, DOPC, DOPE, and DOPS membranes suggests that the spontaneous curvature of monolayers does not affect thyroxin adsorption. It can be assumed that the adsorption plane of the modifier is located between the areas corresponding to the lateral pressure jump in the DOPE and DPhPC membranes and location of charged serine residues in DOPS bilayers.

**Xanthene dyes**

*Table 1* shows that the xanthene dyes discussed in this paper reduce the dipole potential of DPhPC membranes. Evaluation of the effectiveness of the dipole- modifying action of these dyes suggests that Rose Bengal and phloxine B belong to the most effective modifiers; erythrosin belongs to substances with “average” effectiveness; and fluorescein, eosin Y, rhodamine 6G, and rhodamine 101 belong to substances with “low” effectiveness.

When comparing the structures shown in *Table 1*, one can conclude that the type and location of halogen substituents in the dye molecule are the main factors determining the decrease in the membrane dipole potential caused by the introduction of these modifiers. It can be assumed that the pronounced decrease in the dipole potential of membranes caused by the introduction of erythrosine is due to the presence of iodine atoms in its molecule. The lack of the latter in fluorescein or replacement of iodine with bromine in eosin Y results in a loss of the dipole-modifying properties of these compounds. Similarly, replacement of iodine with bromine in phloxine B reduces the effectiveness of this modifier compared to that of Rose Bengal. In this case, the strong dipole-modifying properties of Rose Bengal should be attributed to the presence of chlorine in its structure. The presence of both the iodine and chlorine atoms in the Rose Bengal molecule makes it the most effective dipole modifier among the known xanthene dyes. Replacement of the hydroxyl group in the fluorescein molecule with the amino group in rhodamine molecules has no effect on the modifier’s capability to change the bilayer dipole potential.

Previously, we have shown that the anionic form of the xanthene dye is responsible for the reduction in the membrane dipole potential [44].

Comparing *K *values shows that xanthene dyes are characterized by an order of magnitude higher affinity for phospholipid membranes compared to that of flavonoids and thyroid hormones (*Table 1*).

**Styrylpyridinium dyes**

According to [28], the increase in the dipole potential of DPhPC membranes is directly proportional to the concentration of styrylpyridinium dyes in bathing solutions in a range from 0 to 15 μM. *Table 1* shows the γ value that characterizes the relative change in the membrane dipole potential caused by the introduction of 5 μM of the dye. Based on these results, an efficacy scale of styrylpyridinium dyes can be composed as follows: di-8-ANEPPS have no dipole-modifying effect, RH 160 and RH 237 are characterized by “average” effectiveness, and RH 421 is characterized by the highest effectiveness with respect to the dipole potential of DPhPC membranes. These results for RH dyes are consistent with data presented in [28].

The ability to enhance the φd depends on the orientation and depth of immersion of the dye into the membrane. According to [45], the depth of immersion into the DPhPC bilayer increases in the order RH 160 < RH 421 < RH 237. RH 160 demonstrates minimum immersion into the membrane among the tested dyes, which is probably due to its lowest hydrophobicity. The highest effectiveness of RH 421 with respect to DPhPC membranes given its intermediate adsorption plane can be due to the closest orientation of the long axis of this dye in the membrane to the surface normal [45]. RH 421 and di-8-ANEPPS should have a similar dipole moment, as they have the same length of pyridine complexes. For this reason, the lack of influence of di-8-ANEPPS on the dipole potential of DPhPC membranes can be associated with longer hydrocarbon “tails” compared to those in RH 421, which determine the immersion and orientation of the dye in the bilayer, rather than the structural differences in the pyridine complex.

*Fig. 1B* shows the dependence of increase in the dipole potential of DPhPC, DOPC, DOPE, DOPS, and DOPS/DOPE bilayers on RH 421 concentration in bathing solutions. *Table 2* shows γ values that characterize the relative increase in the membrane dipole potential caused by the introduction of 5 μM of RH 421 in membrane bathing solutions. The dependence of the dipole-modifying action of RH 421 on the type of membrane-forming lipid (DPhPC, DOPC, and DOPS) may indicate the influence of the lateral pressure profile on dye orientation in the bilayer. Furthermore, RH 421 has a low effectiveness with respect to negatively charged DOPS membranes. This may result from the repulsion of negatively charged sulfonate groups of the modifier and serine moieties. In all probability, this contributes to the increase in the positive spontaneous curvature of the monolayer upon adsorption of RH 421 and change in the orientation of the dipole moment of the dye compared to that in DPhPC, DOPC, DOPE, and DOPS/DOPE membranes. The same conclusion was reached in the study of the channel-forming activity of antimicrobial peptides in the presence of RH 421 [15].

## CONCLUSIONS

The dipole-modifying effect of certain flavonoids, steroid muscle relaxants, thyroid hormones, and xanthene and styrylpyridinium dyes on phospholipid bilayers of different compositions was quantitatively characterized. The structural features of the modifiers responsible for their ability to change the value of the membrane dipole potential were identified. Typically, more hydrophobic compounds have more pronounced dipole-modifying properties. In the case of flavonoids, the conformation of the molecule and position of hydroxyl groups are also important, while in the case of xanthene dyes, the important factors are the type and position of halogen substituents. Variation in the phospholipid composition of membranes allowed us to predict the plane of adsorption of the most effective compounds in each group of modifiers. Changing the lateral pressure profile of the bilayer affects the adsorption of phloretin, pancuronium bromide, vecuronium bromide, and RH 421.

## Abbreviations

DPhPC: 1,2-diphytanoyl-sn-glycero-3-phosphocholine
DOPC: 1,2-dioleoyl-sn-glycero-3-phosphocholine
DOPS: 1,2-dioleoyl-sn-glycero-3-phosphoserine
DOPE: 1,2-dioleoyl-sn-glycero-3-phosphoethanolamine
